# Exciton-polaron Rydberg states in monolayer MoSe_2_ and WSe_2_

**DOI:** 10.1038/s41467-021-26304-w

**Published:** 2021-10-21

**Authors:** Erfu Liu, Jeremiah van Baren, Zhengguang Lu, Takashi Taniguchi, Kenji Watanabe, Dmitry Smirnov, Yia-Chung Chang, Chun Hung Lui

**Affiliations:** 1 Department of Physics and Astronomy, University of California, Riverside, CA 92521 USA; 2 National High Magnetic Field Laboratory, Tallahassee, FL 32310 USA; 3 Department of Physics, Florida State University, Tallahassee, FL 32310 USA; 4 International Center for Materials Nanoarchitectonics, National Institute for Materials Science, 1-1 Namiki Tsukuba, Ibaraki, 305-0044 Japan; 5 National Institute for Materials Science, 1-1 Namiki Tsukuba, Ibaraki, 305-0044 Japan; 6 Research Center for Applied Sciences, Academia Sinica, Taipei, 11529 Taiwan

**Keywords:** Electronic properties and materials, Two-dimensional materials

## Abstract

Exciton polaron is a hypothetical many-body quasiparticle that involves an exciton dressed with a polarized electron-hole cloud in the Fermi sea. It has been evoked to explain the excitonic spectra of charged monolayer transition metal dichalcogenides, but the studies were limited to the ground state. Here we measure the reflection and photoluminescence of monolayer MoSe_2_ and WSe_2_ gating devices encapsulated by boron nitride. We observe gate-tunable exciton polarons associated with the 1 s–3 s exciton Rydberg states. The ground and excited exciton polarons exhibit comparable energy redshift (15~30 meV) from their respective bare excitons. The robust excited states contradict the trion picture because the trions are expected to dissociate in the excited states. When the Fermi sea expands, we observe increasingly severe suppression and steep energy shift from low to high exciton-polaron Rydberg states. Their gate-dependent energy shifts go beyond the trion description but match our exciton-polaron theory. Our experiment and theory demonstrate the exciton-polaron nature of both the ground and excited excitonic states in charged monolayer MoSe_2_ and WSe_2_.

## Abstract (web summary)

An exciton polaron is a quasiparticle composed of an exciton dressed with an electron-hole cloud, and this concept has been used to explain the ground excitonic states in charged monolayer transition metal dichalcogenides. Here the authors present experimental and theoretical evidence of exciton-polaron Rydberg states in monolayer MoSe_2_ and WSe_2_.

## Introduction

Hydrogen atoms are known to exhibit the Rydberg spectrum, which played a crucial role in the birth and development of quantum mechanics. As the solid-state counterpart of hydrogen atoms, excitons can also exhibit Rydberg-like optical spectra, which are crucial to revealing the exciton quantum structure. In principle, Rydberg-like spectra can also exist in complex excitonic states strongly coupled to the Fermi sea (FS), despite the lack of experimental evidence. Such spectra are expected to exhibit distinctive characteristics originating from the complex many-body interactions, which can help us understand the nature of coupled states between excitons and FS.

**Fig. 1:**
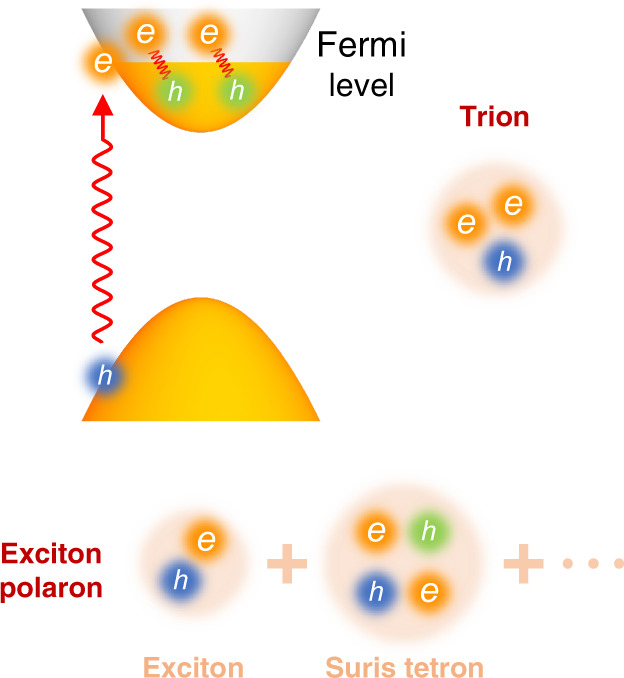
Schematic of trion and exciton polaron. When a photo-generated exciton interacts with the Fermi sea (FS), it can excite many electron–hole pairs on the FS. The trion is a bound state between an exciton and one FS electron. The exciton-polaron state is the linear combination of a bare exciton, an exciton dressed with one FS electron–hole pair (Suris tetron), and higher-order components that represent an exciton dressed with two and more FS electron–hole pairs.

In a conventional scenario of exciton–FS interactions, the exciton captures an extra charge to form a three-body bound state called a trion (Fig. 1), in analogy to the hydrogen ion^1–3^. The trion picture has been widely applied to explain the optical spectra of charged semiconductors, such as quantum wells^4–6^, carbon nanotubes^7^, and two-dimensional (2D) transition metal dichalcogenides^8–11^. Recent research, however, points out that the three-particle picture may be inadequate to account for the complex exciton–FS interactions^12–20^. In a more realistic description, an exciton can excite many electron–hole pairs in the FS (Fig. 1). When the exciton is coupled to the FS electron–hole pairs, it can form a complex quasiparticle called exciton polaron^14–18^ (not the conventional polarons dressed by phonons). The exciton polaron is fundamentally different from the trion. First, the exciton polarons are charge-neutral bosons, whereas the trions are fermions with net electric charge. Second, a general exciton-polaron state is a linear combination of a bare exciton, an exciton dressed with one FS electron–hole pair (called a Suris tetron), and higher-order components that represent an exciton dressed with two and more FS electron–hole pairs (Fig. 1). Such a many-body configuration contrasts with the simple three-particle configuration of the trion.

There have been ongoing debates to distinguish the trion and exciton-polaron pictures, because they give similar energy in the low-charge-density or weak-coupling regime. For instance, a Suris tetron can be viewed as a trion bound with a FS hole (Fig. 1)^12–14^. When the FS is small, the trion–hole binding is weak, and hence the polaron has almost the same binding energy as the trion. However, the trion and polaron binding energies become markedly different under strong exciton–FS coupling, which can be realized in two ways: (1) enlarging the FS (e.g., by electrostatic gating) and (2) increasing the exciton size (e.g., by using excited Rydberg exciton states). It would therefore be interesting to investigate excited excitonic Rydberg states coupled to a tunable FS, but the related experimental studies have been lacking.

In this article, we apply both approaches to investigate the ground and excited excitonic Rydberg states coupled to the gate-tunable FS in monolayer MoSe_2_ and WSe_2_ devices. By using reflectance contrast and photoluminescence (PL) spectroscopy, we observe optical signatures of the exciton-polaron states associated with the 1s–3s Rydberg excitons and characterize their gate-dependent optical properties. We also establish a comprehensive exciton-polaron theory to quantitatively explain our results. Our experiment and theory support the exciton-polaron picture rather than the commonly used trion picture. The observation of exciton-polaron Rydberg states shall significantly enrich the excitonic physics in 2D semiconductors.

## Results and discussion

### Exciton Rydberg states in monolayer MoSe_2_

**Fig. 2:**
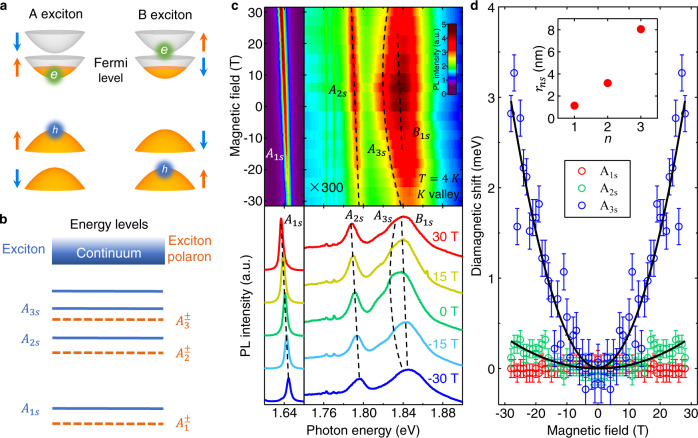
Excitonic states in monolayer MoSe_2_. **a** The band configurations of *A* and *B* bright excitons in monolayer MoSe_2_. The arrows denote the electron spin of the bands. The Fermi level reaches only the inner subbands in our experiment. **b** The internal energy levels of exciton and exciton polaron. **c** Magnetic-field-dependent PL maps of monolayer MoSe_2_ plotted in arbitrary unit (a.u.) at zero gate voltage and temperature *T* = 4 K. The lower panel shows the cross-cut PL spectra at five different magnetic fields. The dashed lines highlight the exciton energy shifts. The PL intensity in 1.74–1.90 eV is magnified 300 times for clarity. **d** The diamagnetic energy shift of the *A*-exciton 1s, 2s, and 3s states (denoted by red, green, and blue circles, respectively) as a function of the magnetic field. The error bars represent the uncertainty in fitting the PL spectra with multiple Lorentzian functions. The lines are quadratic fits. The inset shows the root-mean-square exciton radii (*r*_*n*s_) for the principal quantum number *n* = 1, 2, 3. ${r}_{2{{{{{\rm{s}}}}}}}$ and ${r}_{3{{{{{\rm{s}}}}}}}$ are extracted from the diamagnetic shifts. ${r}_{1{{{{{\rm{s}}}}}}}$ is obtained by a theoretical model and it matches the experimental value in ref. ^27^.

We investigate ultraclean monolayer MoSe_2_ single-gate devices encapsulated by hexagonal boron nitride (BN) and equipped with thin graphite as the contact and back-gate electrodes (see “Methods” and Supplementary Fig. 1). Monolayer MoSe_2_ possesses a direct bandgap in two valleys, where the conduction (valence) band is split into two subbands with ~30 meV (~180 meV) separation by spin–orbit coupling^21^. The inner and outer subbands produce the *A* and *B* bright excitons, respectively (Fig. 2a). Each exciton hosts a series of internal energy levels, analogous to the hydrogenic Rydberg levels (Fig. 2b)^22–28^. We have measured the PL at magnetic fields *B* = −31 T to +31 T and the gate voltage *V*_g_ = 0 V (Fig. 2c). We observe the *A*-exciton and *B*-exciton 1s states (${A}_{1{{{{{\rm{s}}}}}}}$, ${B}_{1{{{{{\rm{s}}}}}}}$) and, between them, the *A*-exciton 2s and 3s states (${A}_{2{{{{{\rm{s}}}}}}}$, ${A}_{3{{{{{\rm{s}}}}}}}$)^26–28^. From the magnetic-field-dependent exciton energies, we extract the linear Zeeman shift and quadratic diamagnetic shift^27–30^. ${A}_{2{{{{{\rm{s}}}}}}}$ and ${A}_{3{{{{{\rm{s}}}}}}}$ excitons exhibit noticeable diamagnetic shift, from which we can derive their root-mean-square radii *r*_2s_ = 3.2 nm and *r*_3s_ = 8.1 nm (Fig. 2d). The *A*_1s_ diamagnetic shift is too small to be discerned in our experiment. But from the extracted *r*_2s_ and *r*_3s_ value, we can apply a model calculation to deduce the *A*_1s_ root-mean-square radius to be *r*_1s_ = 1.1 nm, which matches the experimental value in the literature^27^ (Supplementary Table 1).

### PL and reflectance contrast measurements on monolayer MoSe_2_

**Fig. 3:**
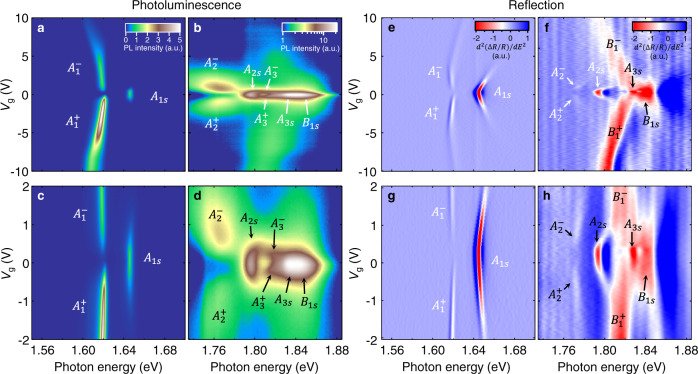
Optical signatures of exciton-polaron Rydberg states in monolayer MoSe_2_. **a**, **b** Gate-dependent PL maps. **c**, **d** Zoom-in PL maps for gate voltages *V*_g_ = −2 to +2 V. **a**, **c** share the same color scale bar; **b**, **d** share the same log-scale color bar. **e**, **f** Gate-dependent maps of the second energy derivative of the reflectance contrast d^2^(Δ*R*/*R*)/d*E*^2^. **g**, **h**, Zoom-in d^2^(Δ*R*/*R*)/d*E*^2^ maps for *V*_g_ = −2 to +2 V. **e**–**h** share the same color scale bar. The intensity of the spectra in **b**, **d** (**f**, **h**) is magnified 300 (20) times. The measurements were made at sample temperature *T* $\approx$ 15 K with no magnetic field. All PL intensity and d^2^(Δ*R*/*R*)/d*E*^2^ values are plotted in arbitrary units (a.u.).

We have measured the gate-dependent reflectance contrast (Δ*R*/*R*) and PL maps of monolayer MoSe_2_, which reveal the absorption and emission properties of the material, respectively (Fig. 3 and Supplementary Figs. 2–4; see “Methods”). We further take the second energy derivative of the reflectance contrast ${{{{{{\rm{d}}}}}}}^{2}(\triangle R/R)/{{{{{\rm{d}}}}}}{E}^{2}$ to sharpen the weak features (Fig. 3e–h). We observe that the ${A}_{1{{{{{\rm{s}}}}}}-3{{{{{\rm{s}}}}}}}$ and ${B}_{1{{{{{\rm{s}}}}}}}$ excitons subside on the electron (−) and hole ($+$) sides and below them emerge new pairs of features labeled as ${A}_{1-3}^{\pm }$ and ${B}_{1}^{\pm }$, respectively (Fig. 3; ${A}_{3}^{\pm }$ are observed only in PL). Our gate-dependent results contrast with and complement the early report of a trion excited state at an unknown doping level in monolayer WS_2_^31^.

${A}_{1-3}^{\pm }$ and ${B}_{1}^{\pm }$ exhibit intriguing gate-dependent optical intensity. We first compare the ${A}_{1}^{\pm }$ and ${B}_{1}^{\pm }$ absorption properties inferred from the second-order differential reflectance contrast maps (Fig. 3e–h). ${A}_{1}^{\pm }$ are suppressed at |*V*| > 4 V. Such gate-induced suppression is widely known, but the underlying mechanism is still uncertain. Here we can gain some insight by comparing ${A}_{1}^{\pm }$ and ${B}_{1}^{\pm }$. ${B}_{1}^{\pm }$ are less suppressed than ${A}_{1}^{\pm }$, and ${B}_{1}^{+}$ remains prominent on the hole side (Fig. 3f). Exciton suppression usually comes from two effects: (1) the plasma screening effect—free charges screen the Coulomb interaction; (2) the state-filling effect—carriers occupy the band-edge states that are needed to form excitons. *A* and *B* excitonic states experience similar screening effects because they share the same dielectric environment. But they have different state-filling effects, because the injected carriers can block the *A* excitons in the inner subbands but not the *B* excitons in the outer subbands (Fig. 2a and Supplementary Fig. 5). Such a difference is more prominent on the hole side than on the electron side due to the large valence-band splitting. Correspondingly, we observe stronger gate-induced suppression on ${A}_{1}^{\pm }$ than on ${B}_{1}^{\pm }$, on the hole side than on the electron side. The state-filling effect should therefore be the major suppression mechanism. Gate-induced suppression is also found in PL, but the *A*–*B* contrast is less pronounced (Fig. 3).

### Gate-dependent properties of the exciton-polaron Rydberg states in monolayer MoSe_2_

**Fig. 4:**
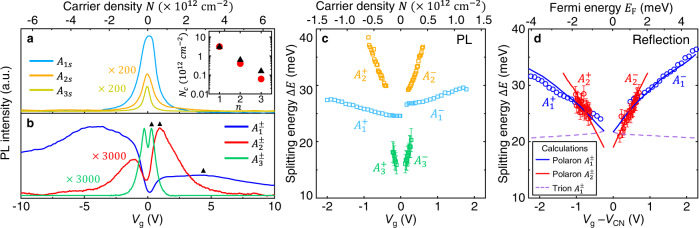
Gate-dependent optical characteristics of ground and excited exciton polarons in monolayer MoSe_2_. **a**, **b** Gate-dependent integrated PL intensity of excitons and exciton polarons, plotted in arbitrary unit (a.u.), as a function of gate voltage (bottom axis) and charge density (top axis). The charge neutrality point is at *V*_g_ = 0 V. We magnify the ${A}_{2s}$, ${A}_{3s}$ (${A}_{2}^{\pm }$, ${A}_{3}^{\pm }$) PL intensity 200 (3000) times for clarity. The inset shows the charge density at maximum PL intensity in **b** (black triangles), compared to the quenching charge density (red dots) expected from the state-filling effect with the exciton radii in Fig. 1d. **c** Gate-dependent PL splitting energy between ${A}_{1}^{\pm }$, ${A}_{2}^{\pm }$, ${A}_{3}^{\pm }$ and ${A}_{1{{{{{\rm{s}}}}}}}$, ${A}_{2{{{{{\rm{s}}}}}}}$, ${A}_{3{{{{{\rm{s}}}}}}}$, respectively (denoted by cyan, orange, and green squares, respectively). **d** Gate-dependent splitting energies between the ${A}_{1}^{\pm }$ and ${A}_{1{{{{{\rm{s}}}}}}}$ absorption peaks (blue circles) and between the ${A}_{2}^{\pm }$ and ${A}_{2{{{{{\rm{s}}}}}}}$ reflection features (red circles). The charge neutrality point is at *V*_CN_ $\approx$ 0.25 V, which has been subtracted from *V*_g_. The purple dashed lines are the calculated trion binding energy. The blue (red) solid lines are the calculated ${A}_{1}^{\pm }$ (${A}_{2}^{\pm }$) binding energies by our theoretical model of exciton polaron. The error bars (not shown if smaller than the symbols) in **c** (**d**) represent the uncertainty in fitting the PL (reflection) features.

We next compare ${A}_{1}^{\pm }$, ${A}_{2}^{\pm }$, and ${A}_{3}^{\pm }$. Figure 4a, b displays the gate-dependent excitonic PL intensity. When the charge density increases, ${A}_{1{{{{{\rm{s}}}}}}}$, ${A}_{2{{{{{\rm{s}}}}}}}$, and ${A}_{3{{{{{\rm{s}}}}}}}$ quickly subside, but ${A}_{1}^{\pm }$, ${A}_{2}^{\pm }$, and ${A}_{3}^{\pm }$ first grow and then diminish. Their respective critical charge density (*N*_c_), defined at maximum PL, decreases monotonically from ${N}_{1}$ $\approx$ 3 × 10^12^ cm^−2^, ${N}_{2}$ $\approx$ 6.2 × 10^11^ cm^−2^ to ${N}_{3}$ $\approx$ 1.6 × 10^11^ cm^−2^ on the electron side (black triangles in Fig. 4a, b); the suppression increases from low to high Rydberg states. The observation can be roughly explained by the increasing state-filling effect from low to high states. High Rydberg excitons have larger spatial size and smaller *k*-space extent than low Rydberg excitons. From our extracted radii of ${A}_{1{{{{{\rm{s}}}}}}}$, ${A}_{2{{{{{\rm{s}}}}}}}$, and ${A}_{3{{{{{\rm{s}}}}}}}$ excitons (inset of Fig. 2d)^27^, we estimate their radii in the *k*-space to be ${k}_{1{{{{{\rm{s}}}}}}}$ $\approx$ 0.44 nm^−1^, ${k}_{2{{{{{\rm{s}}}}}}}$ $\approx$ 0.16 nm^−1^ and ${k}_{3{{{{{\rm{s}}}}}}}$ $\approx$ 0.062 nm^−1^ by the uncertainty relation $\Delta x\Delta k$ $\approx$ 1/2. The corresponding charge density to block the *k*-space of these excitons decreases from ${N}_{1{{{{{\rm{s}}}}}}}$ $\approx$ 3.1 × 10^12^ cm^−2^, ${N}_{2{{{{{\rm{s}}}}}}}$ $\approx$ 3.9 × 10^11^ cm^−2^ to ${N}_{3{{{{{\rm{s}}}}}}}$ $\approx$ 6.1 × 10^10^ cm^−2^ (red dots in the inset of Fig. 4a), when we consider the relation $N={k}^{2}/2\pi$ for 2D quadratic bands with valley degeneracy. These value roughly match our measured critical charge density (inset of Fig. 4a) and hence support that ${A}_{1}^{\pm }$, ${A}_{2}^{\pm }$, ${A}_{3}^{\pm }$ are associated with the ${A}_{1{{{{{\rm{s}}}}}}}$, ${A}_{2{{{{{\rm{s}}}}}}}$, ${A}_{3{{{{{\rm{s}}}}}}}$ excitons, respectively.

In addition to the gate-dependent suppression, the Rydberg states also exhibit remarkable gate-dependent energy shifts. Figure 3c, d displays the splitting energies ($\Delta E$) between ${A}_{1}^{\pm }$, ${A}_{2}^{\pm }$, ${A}_{3}^{\pm }$ and ${A}_{1{{{{{\rm{s}}}}}}}$, ${A}_{2{{{{{\rm{s}}}}}}}$, ${A}_{3{{{{{\rm{s}}}}}}}$, respectively, as a function of gate voltage *V*_g_, charge density *N*, and Fermi energy *E*_F_. Their different splitting energies at *E*_F_ = 0 (i.e., the exciton-polaron binding energy) and their slope with respect to *E*_F_ are summarized in Table 1. The slope of $\Delta E$ increases from low to high Rydberg states, indicating increasing exciton–FS interaction from low to high states.

**Table 1:** Measured binding energies of exciton polarons for monolayer MoSe_2_.

		${A}_{1}^{-}$	${A}_{1}^{+}$	${A}_{2}^{-}$	${A}_{2}^{+}$	${A}_{3}^{-}$	${A}_{3}^{+}$
PL	$\Delta E$ (${E}_{{{{{{\rm{F}}}}}}}=0$)	26.1 ± 0.1	24.3 ± 0.1	24.6 ± 0.2	22.7 ± 0.4	13.0 ± 0.5	13.1 ± 0.3
	${{{{{\rm{d}}}}}}(\Delta E)/{{{{{\rm{d}}}}}}{E}_{{{{{{\rm{F}}}}}}}$	1.10 ± 0.07	−0.75 ± 0.05	11.1 ± 0.6	−7.9 ± 0.6	13.7 ± 2.3	−23.8 ± 1.6
Reflection	$\Delta E$ (${E}_{{{{{{\rm{F}}}}}}}=0$)	23.1 ± 0.2	23.7 ± 0.1	16.4 ± 0.7	22.6 ± 1.0	—	—
	${{{{{\rm{d}}}}}}(\Delta E)/{{{{{\rm{d}}}}}}{E}_{{{{{{\rm{F}}}}}}}$	2.9 ± 0.1	−1.91 ± 0.06	7.0 ± 1.0	−2.9 ± 1.6	—	—

$\Delta E$ (${E}_{{{{{{\rm{F}}}}}}}=0$) denotes the energy separation (in the unit of meV) between the exciton and exciton polaron at the charge neutrality point. ${{{{{\rm{d}}}}}}(\Delta E)/{{{{{\rm{d}}}}}}{E}_{{{{{{\rm{F}}}}}}}$ denotes the slope of $\Delta E$ with respect to the Fermi energy (${E}_{{{{{{\rm{F}}}}}}}$).

### Calculations of exciton polarons for monolayer MoSe_2_

To clarify the nature of ${A}_{1-3}^{\pm }$ states, we have calculated the absorption resonances of ${A}_{1{{{{{\rm{s}}}}}}}$, ${A}_{2{{{{{\rm{s}}}}}}}$, ${A}_{1}^{\pm }$, and ${A}_{2}^{\pm }$ states by first-principles calculations based on both the trion picture and exciton-polaron picture (see the details in Supplementary Note 7). Our calculations apply a realistic long-range Coulomb potential and consider the carrier screening effect, state-filling effect, and the band-structure renormalization by the FS^29^. The calculated ${A}_{1}^{\pm }$ trion binding energy decreases with the gate voltage *V*_g_ due to the increasing charge screening and state-filling effect at increasing carrier density (dashed lines in Fig. 4d). The result contradicts our observation that both the excitonic splitting energies $\Delta {E}_{1}$ and $\Delta {E}_{2}$ increase with carrier density. The trion picture is therefore unable to explain the *E*_F_ dependence of $\Delta {E}_{1}$ and $\Delta {E}_{2}$.

Our calculations based on the exciton-polaron theory produce markedly different results (solid lines in Fig. 4d and Supplementary Figs. 7 and 8). In our theory, we only consider an exciton polaron as the linear combination of a bare exciton and a Suris tetron (Fig. 1 and Supplementary Note 7); a Suris tetron is a four-particle bound state that represents an exciton coupled to one FS electron–hole pair^12, 13^ (Fig. 1). The higher-order components, which represent an exciton coupled to two and more FS electron–hole pairs, are neglected in our model. Such a simplification is justified, because, after an exciton becomes a Suris tetron, its coupling to additional FS electron–hole pairs is much reduced due to screening. The higher-order terms are important only in very high carrier density.

In our theory, there are two branches of exciton polarons with different compositions of the bare exciton and the Suris tetron^12–15^. At the low-charge-density regime, the higher-energy branch is dominated by the bare exciton component and hence exhibits strong oscillator strength; the lower-energy branch is dominated by the Suris tetron component and exhibits weak oscillator strength. As the charge density increases, the exciton component in the lower branch will increase, and the Suris tetron component in the higher branch will increase, leading to a transfer of oscillator strength from the higher to lower branch (Supplementary Figs. 7 and 8).

The energy separation between the two exciton-polaron branches is here defined as the binding energy of the exciton polaron. To compare the binding energies of trion and exciton polaron, we may consider a Suris tetron as a trion bound with a FS hole (in the case of electron FS). In the low-charge-density limit, the trion–hole binding is negligible due to the small phase space of the FS. As a result, the exciton-polaron binding energy (e.g., the energy difference between a bare exciton and a Suris tetron) is reduced to the trion binding energy (Fig. 4d). But when the FS expands, the enlarged phase space of the FS hole increases the trion–hole binding, which contributes to the total polaron binding. In addition, the coupling between the bare exciton and Suris tetron increases as the FS expands, further widening the separation between the two polaron branches^14, 15^. Consequently, the exciton-polaron binding energy increases with the charge density, unlike the trion binding energy that decreases with the charge density.

We have calculated the splitting energy between the two polaron branches (i.e., the polaron binding energy) for both the ground and excited states in monolayer MoSe_2_ (solid lines in Fig. 4d). We note that, for a monolayer semiconductor embedded in BN, a large fraction of the electric field between carriers goes out of the semiconductor plane; this reduces the in-plane free-carrier screening and the reduction of screening increases with the exciton size^22^. Our calculation uses different values of an adjustable parameter *f* to account for the different reduction of screening in the ground and excited states. By using the best-fit value *f* = 0.05 (0.025) for the ground (excited) states, our calculations can quantitatively reproduce the increasing splitting energy with charge density for both the ground and excited states (Fig. 4d and see the details in Supplementary Note 7). The experiment–theory agreement strongly supports the exciton-polaron nature of the states.

### Exciton-polaron Rydberg states in monolayer WSe_2_

**Fig. 5:**
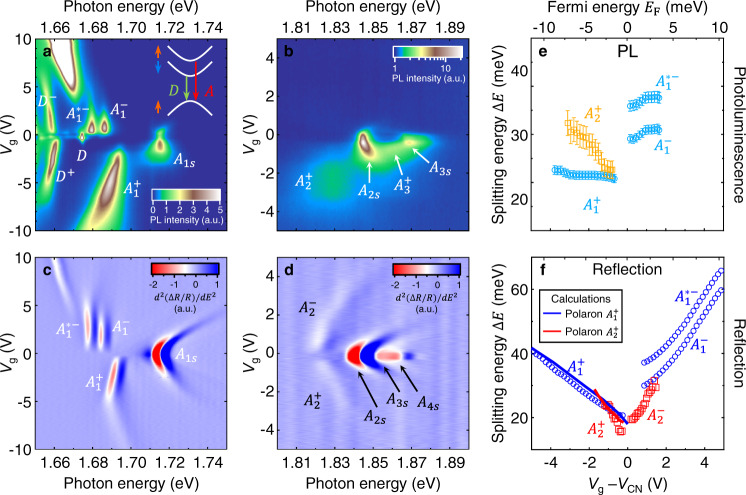
Optical signatures of exciton-polaron Rydberg states in monolayer WSe_2_. **a**, **b** Gate-dependent PL maps plotted in arbitrary unit (a.u.). The inset in **a** shows the band configurations in monolayer WSe_2_. The arrow beside each band denotes its electron spin. The red and green arrows between the bands denote the recombination transitions for bright exciton (*A*) and dark exciton (*D*), respectively. **c**, **d** Gate-dependent maps of the second energy derivative of the reflectance contrast d^2^(Δ*R*/*R*)/d*E*^2^ plotted in arbitrary unit (a.u.). **e** Gate-dependent PL energy separation between ${A}_{1}^{+}$, ${A}_{1}^{-}$, ${A}_{1}^{\ast -}$ and ${A}_{1{{{{{\rm{s}}}}}}}$ (cyan circles) and between ${A}_{2}^{+}$ and ${A}_{2{{{{{\rm{s}}}}}}}$ (orange squares) in **a**, **b**. The error bars (not shown if smaller than the symbols) represent the uncertainty in fitting the PL spectra with multiple Lorentzian functions. **f** Gate-dependent energy separation between ${A}_{1}^{+}$, ${A}_{1}^{-}$, ${A}_{1}^{\ast -}$ and ${A}_{1{{{{{\rm{s}}}}}}}$ (blue circles) and between ${A}_{2}^{\pm }$ and ${A}_{2{{{{{\rm{s}}}}}}}$ (red squares) in the d^2^(Δ*R*/*R*)/d*E*^2^ maps in **c**, **d**. We subtract the charge-neutrality gate voltage *V*_CN_ $\approx$ −0.14 V from the applied gate voltage *V*_g_. The blue (red) solid line is the calculated ${A}_{1}^{+}$ (${A}_{2}^{+}$) binding energy by our theoretical model of exciton polarons. The measurements were made at temperature *T* $\approx$ 15 K with no magnetic field.

We have also observed exciton-polaron Rydberg states in monolayer WSe_2_. Figure 5a–d displays the gate-dependent PL maps and second-order differential reflectance contrast maps of a BN-encapsulated monolayer WSe_2_ device. The results reveal the 1s–4s exciton Rydberg states in the charge neutrality region^27–30, 32, 33^. As we tune the Fermi level away from charge neutrality, we observe the exciton polarons (${A}_{1}^{\pm }$, ${A}_{2}^{\pm }$) associated with the 1s and 2s exciton states, as well as a somewhat obscured PL signature of the polaron (${A}_{3}^{+}$) associated with the 3s exciton state.

Compared to monolayer MoSe_2_, the exciton polarons in monolayer WSe_2_ exhibit one distinct feature. That is, in monolayer WSe_2_, while the exciton polarons appear on both the electron and hole sides of the reflection data, the excited-state polarons appear only on the hole side of the PL data (Fig. 5a–d). The mechanism of such an electron–hole asymmetry of excited polarons is unknown at this stage. We speculate that it is related to the opposite spin configuration of the conduction bands in monolayer WSe_2_ compared to that in monolayer MoSe_2_ (Fig. 2a and inset of Fig. 5a). Prior studies^34–42^ have shown that the opposite spin of conduction bands in monolayer WSe_2_ give rise to dark excitons ($D$), phonon replicas, and multiple electron-side exciton polarons (e.g., ${A}_{1}^{\ast -}$, ${A}_{1}^{-}$; Fig. 4a, c), which are not found in monolayer MoSe_2_.

Figure 5e–f displays the splitting energies ($\Delta E$) between ${A}_{1{{{{{\rm{s}}}}}}}$ and ${A}_{1}^{\pm }$ and between ${A}_{2{{{{{\rm{s}}}}}}}$ and ${A}_{2}^{\pm }$ at varying gate voltage and Fermi energy (${E}_{{{{{{\rm{F}}}}}}}$). Their splitting energies at *E*_F_ = 0 (i.e., the exciton-polaron binding energy) and the *E*_F_-dependent slope of $\Delta E$ are summarized in Table 2. The $\Delta E$ slopes of excited polaron states are considerably larger than those of the ground states, consistent with our observation in monolayer MoSe_2_.

**Table 2:** Measured binding energies of exciton polarons for monolayer WSe_2_.

		${A}_{1}^{-}$	${A}_{1}^{\ast -}$	${A}_{1}^{+}$	${A}_{2}^{-}$	${A}_{2}^{+}$
PL	$\Delta E$ (${E}_{{{{{{\rm{F}}}}}}}=0$)	29.0 ± 0.1	35.8 ± 0.1	20.5 ± 0.1	—	18.4 ± 0.3
	${{{{{\rm{d}}}}}}(\Delta E)/{{{{{\rm{d}}}}}}{E}_{{{{{{\rm{F}}}}}}}$	1.2 ± 0.1	1.2 ± 0.1	−0.18 ± 0.02	—	−1.8 ± 0.1
Reflection	$\Delta E$ (${E}_{{{{{{\rm{F}}}}}}}=0$)	27.1 ± 0.1	34.7 ± 0.1	17.5 ± 0.1	17.8 ± 0.4	9.2 ± 0.7
	${{{{{\rm{d}}}}}}(\Delta E)/d{E}_{{{{{{\rm{F}}}}}}}$	2.3 ± 0.1	1.9 ± 0.1	−1.74 ± 0.03	4.7 ± 0.3	−5.5 ± 0.4

$\Delta E$ (${E}_{{{{{{\rm{F}}}}}}}=0$) denotes the energy separation (in the unit of meV) between the exciton and exciton polaron at the charge neutrality point. ${{{{{\rm{d}}}}}}(\Delta E)/{{{{{\rm{d}}}}}}{E}_{{{{{{\rm{F}}}}}}}$ denotes the slope of $\Delta E$ with respect to the Fermi energy (${E}_{{{{{{\rm{F}}}}}}}$).

We have calculated $\Delta E$ on the hole side for monolayer WSe_2_ by using the same exciton-polaron theory as in monolayer MoSe_2_ but with different material parameters and different best-fit values of the screening reduction parameter (*f*) (see the details in Supplementary Note 7). The calculated $\Delta E$ (lines in Fig. 5f) matches reasonably the experimental $\Delta E$ for both the ground and excited states. The results strongly support the exciton-polaron nature of the states.

In our presentation above, we have distinguished the trion and exciton-polaron pictures by considering the different carrier-density dependence of their binding energy. Actually, our theory also predicts markedly different oscillator strength for the trion and exciton polaron. In a simple explanation, let us consider the creation of a trion (with center-of-mass wave vector **k**) from a photo-generated electron–hole pair in the presence of a free carrier (with the same **k**) in a system with translational symmetry. In this case, the initial free-carrier state has one single **k** value. But once this carrier enters into the trion, its wave vector will be dispersed over a wide range of **k** values. The overlap between these two configurations is negligible. As a result, our theory predicts essentially zero oscillator strength for the trion. In comparison, let us consider the creation of an exciton polaron in the presence of a finite FS. In this case, the exciton polaron consists of a bare-exciton component and a Suris-tetron component. The bare-exciton component will bestow the polaron with finite oscillator strength. Therefore, the exciton-polaron model is more reasonable than the trion model to explain the strong optical signals observed in our experiment.

In summary, we have observed gate-tunable exciton-polaron Rydberg states associated with the 1s–3s excitons in monolayer MoSe_2_ and WSe_2_ devices. Our experimental results and theoretical calculations support the exciton-polaron nature of both the ground and excited excitonic states in charged monolayer MoSe_2_ and WSe_2_. We expect that exciton polarons also exist in some other 2D semiconductors with finite charge density. Our findings shall motivate further exploration of exciton polarons and complex many-body interactions in different 2D semiconductors.

Note added in proof: We were aware of two related papers on the topic of exciton polarons (ref. ^43,44^).

## Methods

### Device fabrication

We fabricate monolayer MoSe_2_ and WSe_2_ devices encapsulated by hexagonal BN by stacking different component 2D materials together. We first exfoliate monolayers MoSe_2_ or WSe_2_, multilayer graphene, and thin BN flakes from their bulk crystals onto the Si/SiO_2_ substrates (The MoSe_2_ and WSe_2_ crystals were bought from HQ Graphene Inc.). Afterward, we apply the polycarbonate-based dry-transfer technique to stack them together^45, 46^. In this method, we use a stamp to first pick up a BN flake and then use the BN flake to sequentially pick up two pieces of multilayer graphene (as the contact electrodes), a MoSe_2_ (or WSe_2_) monolayer, a BN thin layer (as the bottom gate dielectric), and a graphene multilayer (as the back-gate electrode). Our method ensures that the MoSe_2_ (or WSe_2_) layer does not contact the polymers during the whole fabrication process, so as to reduce the contaminants and bubbles on the interfaces^47^. Standard electron beam lithography is then applied to pattern and deposit the gold contacts (100-nm thickness). Finally, we anneal the devices at 300 °C for 3 h in an argon environment. Supplementary Fig. 1 shows the schematic and optical image of a representative BN-encapsulated monolayer MoSe_2_ device.

### Determination of carrier density and Fermi energy

We measure the BN thickness in our devices by atomic force microscopy (AFM). For the monolayer MoSe_2_ device in Figs. 2–4, the thickness for the bottom BN is ~26 nm. The injected charge density (*N*) is calculated with the formula *Ne* = *CV*_g_, where $C=\varepsilon {\varepsilon }_{0}/d$ is the capacitance, *d* is the thickness of the bottom BN, $\varepsilon =3.1$ is the dielectric constant of BN^48^, and ${\varepsilon }_{0}$ is the permittivity of free space. We convert the gate voltage into the Fermi energy by using the relation ${E}_{{{{{{\rm{F}}}}}}}={\hslash }^{2}\pi n/{m}_{{{{{{\rm{e}}}}}},{{{{{\rm{h}}}}}}}$, where ${m}_{{{{{{\rm{e}}}}}}}$ (${m}_{{{{{{\rm{h}}}}}}}$) is the electron (hole) effective mass. We adopt ${m}_{{{{{{\rm{e}}}}}}}=0.88{m}_{0}$ and ${m}_{{{{{{\rm{h}}}}}}}=0.74{m}_{0}$ (${m}_{0}$ is the free electron mass) for monolayer MoSe_2_ by fitting our diamagnetic shift with our model calculation. These effective masses are roughly consistent with prior studies^49^. In our experiment on monolayer MoSe_2_, the Fermi level shifts with the gate voltage with a slope of d*E*_F_/d*V*_g_ ≈ 1.80 meV/V (2.13 meV/V) on the electron (hole) side.

For the monolayer WSe_2_ device in Fig. 5, the thickness of the bottom BN is ~40 nm. We use the effective masses ${m}_{{{{{{\rm{e}}}}}}}=0.46{m}_{0}$ for the lower conduction band, ${m}_{{{{{{\rm{e}}}}}}}=0.38{m}_{0}$ for the upper conduction band, and ${m}_{{{{{{\rm{h}}}}}}}=0.42{m}_{0}$ for the top valance band^8^. The Fermi level as a function of gate voltage has a slope of d*E*_F_/d*V*_g_ ≈ 2.23 meV/V (2.44 meV/V) on the electron (hole) side.

### Experimental methods

The optical experiments with no magnetic field (Figs. 3–5) were performed in our laboratory at the University of California, Riverside. We mount the devices in a cryostat (Montana Instruments) with sample temperature at *T* $\approx$ 15 T. For the reflectance contrast measurements, we focus the white light from a broadband light source (Thorlabs, SLS201L) onto the sample with a spot diameter of ~2 μm. We measure the reflected spectrum (*R*_s_) from the monolayer MoSe_2_ (or WSe_2_) sample on the BN/MoSe_2_/BN/Gr/SiO_2_/Si stack and a reference spectrum (*R*_r_) on a nearby area without MoSe_2_ on the BN/BN/Gr/SiO_2_/Si stack. The reflectance contrast (Δ*R*/*R*) is obtained as Δ*R*/*R* = (*R*_s_ − *R*_r_)/*R*_r_. We further take the second energy derivative of the Δ*R*/*R* spectrum to sharpen the weak features.

For the PL experiment with no magnetic field, we excite the samples with a 532-nm continuous laser (Torus 532, Laser Quantum). The laser beam is focused through a microscope objective (NA = 0.6) onto the sample with a spot diameter of ~1 μm. The PL is collected through the same objective in a backscattering geometry and analyzed by a high-resolution spectrometer (Princeton Instruments, IsoPlane 320) with a charge-coupled-device (CCD) camera.

The PL experiments with magnetic field were performed in the National High Magnetic Field Laboratory (NHMFL) in Florida, USA. We use a 31-Tesla DC magnet and a fiber-based probe (the same set-up as in ref. ^29^). The sample temperature is *T* =4 K. A 532-nm continuous laser is directed through a single-mode optical fiber and focused by a lens (NA = 0.67) onto the sample. The sample is mounted on a three-dimensional Attocube piezoelectric translational stage. The PL is collected through a 50/50 beam splitter into a multimode optical fiber and subsequently measured by a spectrometer (Princeton Instruments, IsoPlane 320) with a CCD camera.

## Supplementary information

Supplementary Information

## Data Availability

The data that support the findings of this study are available from the corresponding authors upon reasonable request.
