# Supplementary material for: Exciton-polaron Rydberg states in monolayer MoSe2 and WSe2
Source: Nat Commun. 2021 Oct 21;12:6131. doi: 10.1038/s41467-021-26304-w (PMC8531338; doi:10.1038/s41467-021-26304-w)
Supplement: Supplementary file 1 — Supplementary Information [file 41467_2021_26304_MOESM1_ESM.pdf]

Supplementary Information for

"Exciton-polaron Rydberg states in monolayer $MoSe_2$ and WSe_2"

Erfu Liu[1†], Jeremiah van Baren[1†], Zhengguang Lu[2,3], Takashi Taniguchi[4], Kenji Watanabe[5], Dmitry Smirnov[2], Yia-Chung Chang[6*], Chun Hung Lui[1*]

[1] Department of Physics and Astronomy, University of California, Riverside, California 92521, USA

[2] National High Magnetic Field Laboratory, Tallahassee, Florida 32310, USA

[3] Department of Physics, Florida State University, Tallahassee, Florida 32310, USA

[4] International Center for Materials Nanoarchitectonics, National Institute for Materials Science, 1-1 Namiki Tsukuba, Ibaraki 305-0044, Japan.

[5] National Institute for Materials Science, 1-1 Namiki Tsukuba, Ibaraki 305-0044, Japan.

[6] Research Center for Applied Sciences, Academia Sinica, Taipei 11529, Taiwan

[†] Equal contribution

[*] Corresponding author. Email: yiachang@gate.sinica.edu.tw; joshua.lui@ucr.edu;

Table of contents

Supplementary Note 1: Device schematic and optical image

Supplementary Note 2: Reflectance contrast and the extraction of absorption energies

Supplementary Note 3: Fitting of PL spectra for the high-lying excitonic states

Supplementary Note 4: Calculation of exciton binding energies for monolayer $MoSe_2$

Supplementary Note 5: Comparison of A and B excitonic states in monolayer $MoSe_2$

Supplementary Note 6: Gate-dependent reflection and PL energies

Supplementary Note 7: Theoretical modeling of exciton polarons

7.1. The ground-state exciton polarons

7.2. The excited-state exciton polarons

7.3. Absorption spectra of exciton polarons

7.4. Summary of empirical parameters and experimental features explained

Supplementary Note 1: Device schematic and optical image

We have fabricated monolayer $MoSe_2$ devices encapsulated by hexagonal boron nitride (BN). Supplementary Figure 1 shows the schematic and optical image of a representative device.

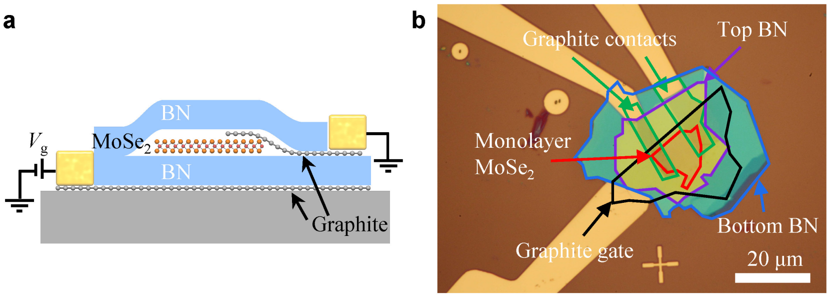

Supplementary Figure 1. Device schematic and optical image. a, Schematic and **b**, optical image of a monolayer $MoSe_2$ device encapsulated by boron nitride (BN). The thickness of the bottom BN flake is between 20 and 30 nm. Two thin graphite flakes are used as contact and gate electrodes to enhance the device performance.

Supplementary Note 2: Reflectance contrast and the extraction of absorption energies

Figure 2e-h of the main paper display the gate-dependent maps of the second energy derivative of reflectance contrast. Here in Supplementary Fig. 2, we show the gate-dependent maps of the reflectance contrast $\Delta R/R$, the first and second energy derivative of $\Delta R/R$. As shown in the comparison, the differentiation method is effective to reveal the weak features.

Our measured reflectance contrast ($\Delta R/R$) spectra mix the real and imaginary parts of the conductivity of monolayer $MoSe_2$ through the optical interference in the $BN/MoSe_2/BN/graphite/SiO_2/Si$ heterostructure (Supplementary Fig. 1). For the spectra of the low-lying states (A_{1s}, $A_1^{\pm}$), as their signals are strong, we can extract the real part of conductivity by solving the optical problem in our stacked material system with the transfer matrix method in linear optics[1]. Our method of extracting the conductivity is similar to the Kramers–Kronig constrained variational analysis developed by A. B. Kuzmenko[2]. For the calculation, we have measured the thickness of top BN, bottom BN, and graphite electrodes in our device by atomic force microscopy (AFM). We adopt the wavelength-dependent complex refractive indices ($\tilde{n}$) of these materials from the literature[3]. The refractive index of BN is taken to be $n_{\text{BN}} = 2.13$ for the frequency range of our experiment; it is consistent with the range $2.0 < n_{\text{BN}} < 2.3$ generally reported in the literature[4, 5]. After we extract the real part of conductivity, we identify the absorption resonance energies of A_{1s} and $A_1^{\pm}$. The splitting energy between A_{1s} and $A_1^{\pm}$ is shown in Fig. 4d.

For the spectra of high-lying states (A_{2s}, $A_2^{\pm}$), as their signals are weak, the extraction of conductivity is inaccurate. Therefore, we directly identify their resonance

energy from the reflection data. Such identification may be subject to a small offset error. But the offset should be largely canceled when we take the difference between the A_{2s} and $A_2^{\pm}$ energies to obtain their energy separation (Fig. 4d).

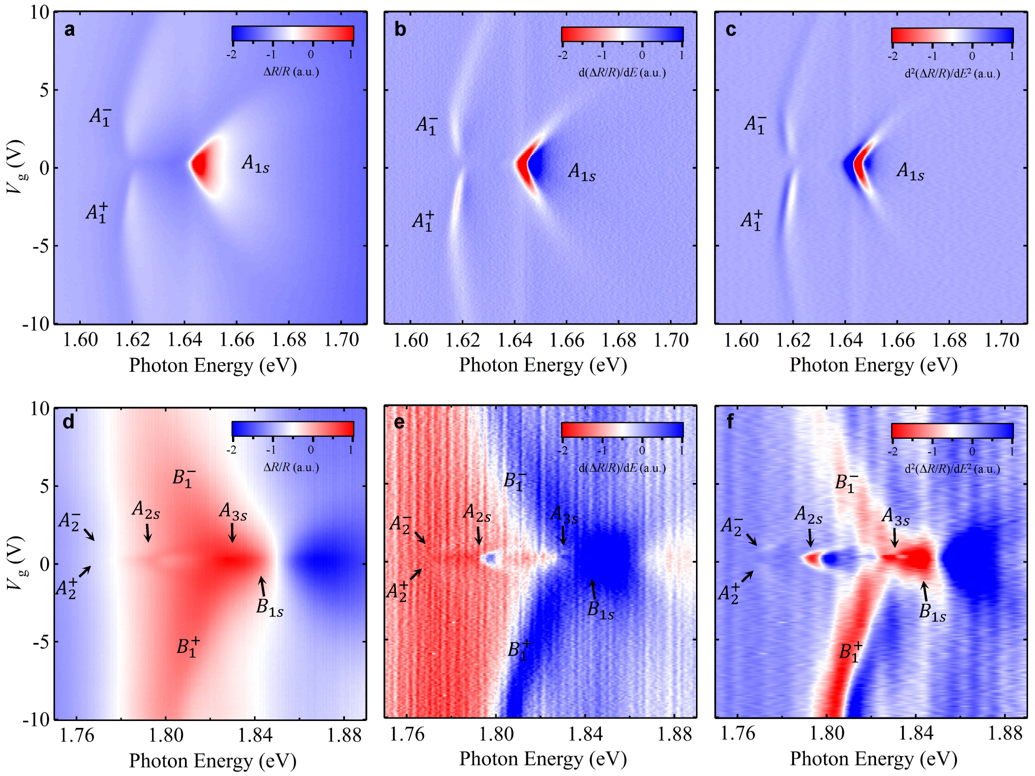

Supplementary Figure 2. Gate-dependent maps of reflection properties. The gate-dependent maps of the reflectance contrast $\Delta R/R$ (**a**, **d**), the first energy derivative of reflectance contrast $d(\Delta R/R)/dE$ (**b**, **e**), and the second energy derivative of reflectance contrast $d^2(\Delta R/R)/dE^2$ (**c**, **f**) for the BN-encapsulated monolayer $MoSe_2$ device used in Figs. 2-4 of the main paper.

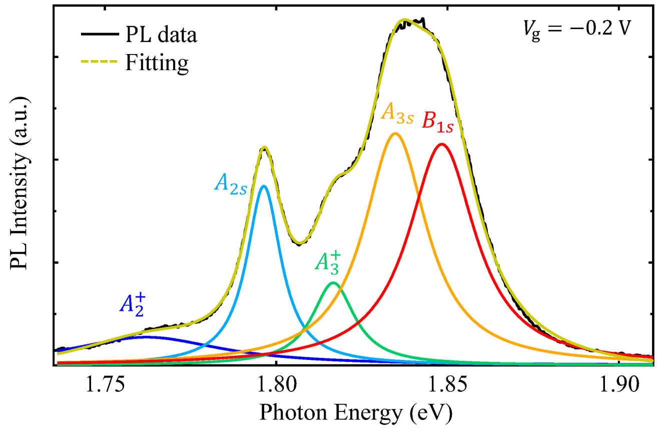

Supplementary Figure 3. Fitting of a representative PL spectrum of the high-lying excitonic states in monolayer $MoSe_2$. The gate voltage is -0.2 V.

Supplementary Note 3: Fitting of PL spectra for the high-lying excitonic states

We have fitted the photoluminescence (PL) spectra of the high-lying excitonic states with multiple Lorentzian functions to extract their emission energies. Supplementary Figure 3 displays the fitting of a representative PL spectrum.

Supplementary Figure 4 shows the PL spectra at different gate voltages. By fitting these PL spectra, we extract the energies of different peaks at different gate voltages (the dashed lines in Supplementary Fig. 4).

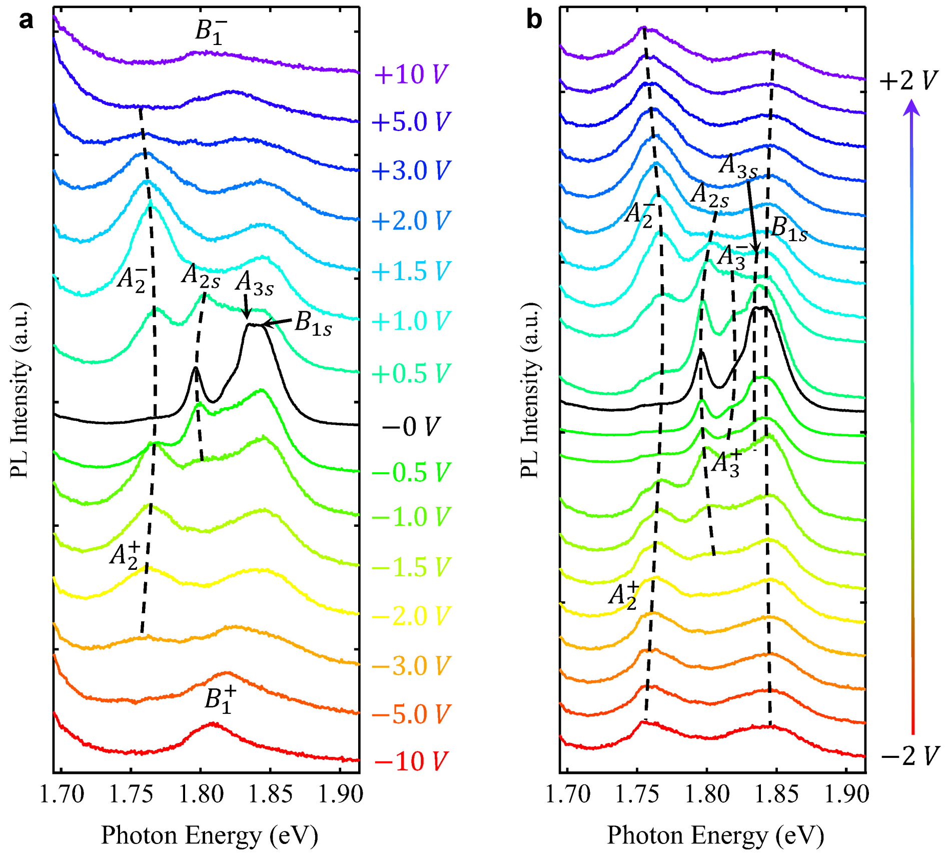

Supplementary Figure 4. PL spectra of monolayer $MoSe_2$ at different gate voltages. a, Cross-cut spectra over a wide range of gate voltages from the PL map in Figure 3b of the main paper. **b**, Cross-cut spectra over a narrow range of gate voltages (0.2 V increment per spectrum) from the PL map in Figure 3d of the main paper. The dashed lines highlight the fitted peak energies of different excitonic states.

Supplementary Note 4: Calculation of exciton binding energies for monolayer $MoSe_2$

Recent research has observed the A-exciton Rydberg states in different monolayer transition metal dichalcogenides (TMDs)[6-9]. Their energies and diamagnetic shifts can be described well by an effective-mass model with the Keldysh potential:[10]

$$V(r) = -\frac{e^2}{8\varepsilon_0 r_0}\left[H_0\left(\frac{\kappa r}{r_0}\right) - Y_0\left(\frac{\kappa r}{r_0}\right)\right], \qquad (1)$$

where H_0 and Y_0 are the Struve and Bessel functions of the second kind, respectively. The Fourier transform of $V(r)$ is:

$$V_0(q) = \frac{e^2}{2\kappa\varepsilon_0 q(1+q\rho_0)}. \quad (2)$$

Here a_B is the Bohr radius of hydrogen atom; κ is the static dielectric constant of the TMD; $\rho_0 = r_0/\kappa$ is a characteristic length of the potential.

For our case of BN-encapsulated monolayer $MoSe_2$, we use $\kappa = 4.4$, $\rho_0 = 20.22$ bohr $= 1.07$ nm, an electron effective mass of $m_e = 0.74m_0$, and a hole effective mass of $m_h = 0.88m_0$ (m_0 is the free electron mass). We calculate the energies of the 1s – 3s A-exciton Rydberg states as $E_{1s} = -212.53$ meV, $E_{2s} = -61.75$ meV and $E_{3s} = -28.98$ meV (we set the band edge energy as zero). Our calculation includes the electron-hole exchange interaction, which is modeled by a contact potential of the form[8] $D(0)\delta(\mathbf{r})$ with

$$D(\mathbf{0}) = \frac{1}{L_c}\sum_{\mathbf{G}\neq\mathbf{0}} \frac{4\pi e^2}{|\mathbf{G}|^2} \left|\langle \tilde{c}_{\uparrow,\mathbf{k}}| e^{i\mathbf{G}\cdot\mathbf{r}} | v^h_{\uparrow,\mathbf{k}}\rangle\right|^2 \quad \text{(evaluated at } \mathbf{k} = 0\text{)}. \quad (3)$$

Here L_c denotes the out-of-plane length of the supercell used in our density functional theory (DFT) calculation. Our calculation shows that the electron-hole exchange interaction reduces the A-exciton binding energy by ~8 meV. We have also calculated the root-mean-square radii of the Rydberg excitons. The results are shown in Supplementary Table 1.

Exciton state	Calculated binding energy (meV)	Calculated radius (nm)	Measured radius (nm)
A_{1s}	212.53	1.13	1.1 (Ref. 7)
A_{2s}	61.75	3.94	3.2
A_{3s}	28.98	8.25	8.1

Supplementary Table 1. Binding energies and root-mean-square radii for the A-exciton Rydberg states in monolayer $MoSe_2$. The measured A_{1s} radius is taken from Ref. 7.

Supplementary Note 5: Comparison of A and B excitonic states in monolayer $MoSe_2$

Figure 3 of the main paper shows that the A exciton polarons are suppressed more strongly by charge injection than the B exciton polarons in monolayer $MoSe_2$. These different behaviors come from their different band configurations (Supplementary Fig. 5). The bright A excitons consist of electrons in the lower conduction band and holes in the higher valence band. When the electrons (holes) are injected into $MoSe_2$, they occupy the states in the lower conduction band (higher valence band), thus suppressing the formation of A exciton. But the bright B excitons consist of electrons in the higher conduction band and holes in the lower valence band. As these states are not occupied by the injected carriers, the B excitons experience no state-filling effect. On the other hand, the A and B excitons and exciton polarons experience very similar screening effect because they share the same dielectric environment. As the A exciton polarons are suppressed more strongly

by charge inection than the B exciton polarons (Fig. 3), we deduce that the major suppression mechanism is the state-filling effect, not the plasma screening effect.

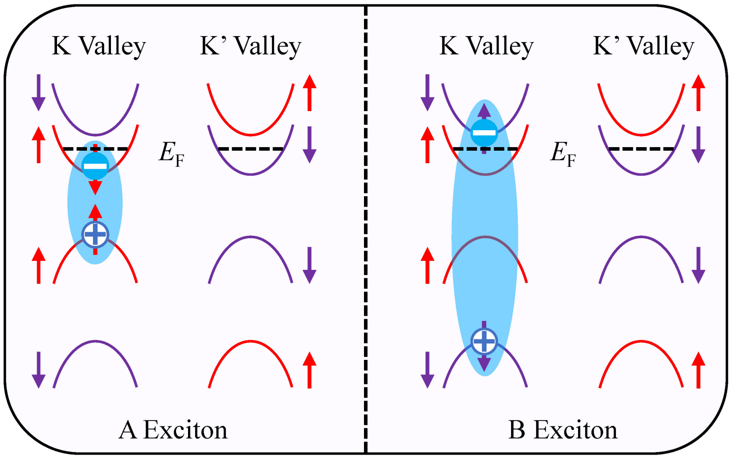

Supplementary Figure 5. Band configurations of the A and B bright excitons in monolayer $MoSe_2$. The dashed lines denote the Fermi level (E_F). The filling of the band-edge states only blocks the formation of the A bright excitons, not the B bright excitons.

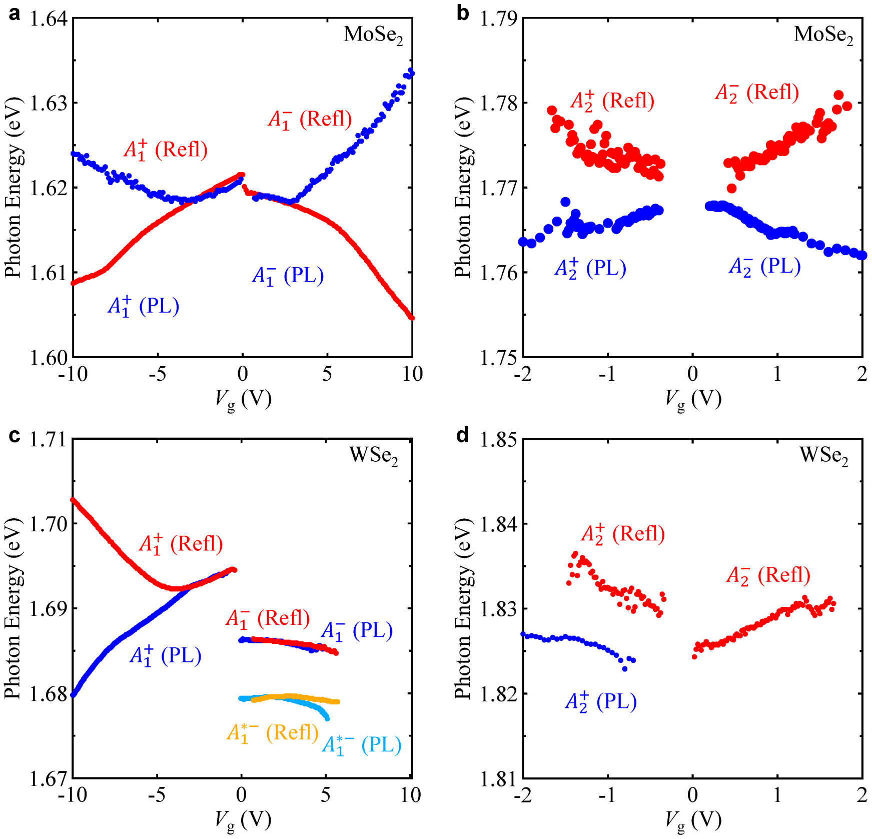

Supplementary Figure 6. Gate-dependent excitonic reflection and PL energies. a, **b**, Extracted energies of different excitonic reflection and PL features for monolayer $MoSe_2$ from Fig. 3 as a function of gate voltage. **c**, **d**, Similar plots for monolayer WSe_2 from Fig. 5.

Supplementary Note 6: Gate-dependent reflection and PL energies

Figure 3 and 5 in the main paper display the gate-dependent maps of PL and second energy derivative of reflectance contrast for monolayer $MoSe_2$ and WSe_2, respectively. Here in Supplementary Figure 6, we show the extracted energies of the excitonic PL and reflection features as a function of gate voltage.

Supplementary Note 7: Theoretical modeling of exciton polarons

7.1. The ground-state exciton polarons

When a photo-generated exciton (either in the ground or excited state) interacts with the Fermi sea (FS), it can couple with many electron-hole pair excitations on the FS to form an exciton polaron. In general, an exciton polaron state is the linear combination of a bare exciton, an exciton dressed with one FS electron-hole pair (called a Suris tetron[11, 12]), and higher-order components that represent an exciton dressed with two and more FS electron-hole pairs. The Suris tetron can be considered as the bound state between a trion and a FS hole. In our model, we only include the bare-exciton and Suris-tetron components, and neglect the higher-order components (here we call it the tetron model). Such an approximation is reasonable when the Fermi energy (E_F) is much smaller than the exciton-polaron binding energy (E_B). In our experiment, E_B increases from ~20 meV to ~60 meV when E_F goes from 0 to ~20 meV. The tetron model should be good to explain the 2s features, which only appear at small E_F (a few meV), and still be decent to explain the 1s features, which appear from E_F = 0 ~ 20 mV.

In our calculation, we consider the interaction between two carriers in the form of the Keldysh potential. When an exciton Rydberg state (1s, 2s, …) interacts with the FS, an electron in the FS will be scattered from a state at wave vector $\mathbf{k}_1$ inside the FS ($|\mathbf{k}_1| < k_F$) into another state at wave vector $\mathbf{k}_2$ outside the FS ($|\mathbf{k}_2| > k_F$). Here k_F is the magnitude of the Fermi wave vector $\mathbf{k}_F$, which is related to the Fermi energy E_F via the relation $E_F = \hbar^2 k_F^2/2m_c$; m_c is the effective carrier mass ($m_c = m_e$ and m_h for electron and hole, respectively). The difference $\mathbf{q} = \mathbf{k}_2 - \mathbf{k}_1$ will be compensated by a recoil of the exciton center-of-mass (CM) motion with wave vector $-\mathbf{q}$. So, the total momentum of the system remains zero. As a result, the kinetic energy (K) of the system increases by:

$$K(\mathbf{k}_1, \mathbf{k}_2) = \frac{\hbar^2 |\mathbf{k}_2 - \mathbf{k}_1|^2}{2M_X} + \frac{\hbar^2 k_2^2}{2m_c} - \frac{\hbar^2 k_1^2}{2m_c}, \tag{4}$$

where $M_X = m_e + m_h$ is the total effective mass of the exciton. The increase of kinetic energy will be overcome by the potential energy as a result of scattering between the electrons and the valence holes. Such scattering allows the formation of an exciton-polaron bound state, which has been studied for the 1s exciton state in the literature[13-17]. The spectral density of the system consists of a peak at energy $-E_B$ (E_B is the binding energy of the exciton polaron) with respect to the energy of the non-interacting system, whose energy is described by $E_{1s} + E_{FS}$, *i.e.* the 1s exciton energy plus the total energy of a rigid FS. In addition, there is a continuum of states with energies $K(\mathbf{k}_1, \mathbf{k}_2) + E_{1s} + E_{FS}$, which correspond to the unbound exciton-polaron states associated with the 1s exciton. In the limit of just one electron in the FS, the 1s exciton polaron reduces to the trion state. In this case, the continuum of unbound trion states is

characterized by the energy $K(\mathbf{k}_2) + E_{1s}$, where

$$K(\mathbf{k}_2) = \frac{\hbar^2 k_2^2}{2M_X} + \frac{\hbar^2 k_2^2}{2m_c} = \frac{\hbar^2 k_2^2}{2\mu_T} \tag{5}$$

with μ_T being the trion reduced mass.

7.2. The excited-state exciton polarons

The 2s exciton state can also interact with the FS to form a quasi-bound state, which sits in the continuum of 1s-associated exciton polarons with varying CM momenta. The coupled state is quasi-bound because it is unlikely for the 2s exciton polaron to get a large enough binding energy E_{B2} such that $E_{2s} + E_{FS} - E_{B2}$ is below $E_{1s} + E_{FS}$ (the threshold of the continuum of 1s-associated exciton polarons). In the limit of just one electron in the FS, the 2s exciton level sits in the unbound 1s-associated trion continuum. Thus, a trion bound state formed by a 2s exciton and an electron is not well defined. However, when there is a sufficient number of electrons in the FS, a 2s-associated exciton polaron can still exist as a resonance feature. Therefore, to study the 2s-associated exciton polaron, one must also consider its interaction with the continuum of 1s-associated exciton polarons.

A simple way to describe the above scenario is to simulate the continuum of the FS polarization by a large set of trial wave functions for all the particles in the tetron model. Ref. 13 has conducted such calculations for the case of a tetron with infinite valence hole mass. Here we adopt a similar strategy but extend the theory to the case of finite valence hole mass. The Hamiltonian of the exciton polaron can be represented by a matrix in the chosen basis set. If the basis set is sufficiently large and flexible, the eigenstates of the finite matrix can capture the essential physics of the exciton polaron system, including the interaction between a 2s-associated exciton polaron and the continuum of 1s-associated exciton polarons.

For the bare exciton component, the basis functions take the form

$$\Phi^X_{n_1,m_1}(\mathbf{r}_1, \mathbf{r}_h) = f_{n_1,m_1}(\mathbf{r}_{1h}) e^{\mathrm{i}\mathbf{Q}\cdot\mathbf{R}_X}. \tag{6}$$

For the Suris-tetron component, we choose a basis set that contains the product of envelope functions:

$$\Phi_{n_1,n_2,n_3,m_1,m_2,m_3}(\mathbf{r}_1, \mathbf{r}_2, \mathbf{r}_3, \mathbf{r}_h) = f_{n_1,m_1}(\mathbf{r}_{1h}) f_{n_2,m_2}(\mathbf{r}_{2h}) f_{n_3,m_3}(\mathbf{r}_{3h}) e^{\mathrm{i}\mathbf{Q}\cdot\mathbf{R}_{CM}}. \tag{7}$$

Here $f_{n,m}(\mathbf{r}_{ij}) = r_{ij}^s e^{\mathrm{i}m\varphi_{ij}} e^{-\alpha_n r_{ij}}$ ($s = 1$ for $m \neq 0$ and $s = 0$ for $m = 0$) for $\mathbf{r}_{1h}$ and $\mathbf{r}_{2h}$; the two electrons above the FS are labeled by 1 and 2; the missing electron (a FS hole) in the FS is labeled by 3; the valence hole is labeled by h. $f_{n,m}(\mathbf{r}_{ij})$ describes the correlation function between particles i and j. The lack of direct correlation in the coordinates $\mathbf{r}_{12}$, $\mathbf{r}_{13}$ and $\mathbf{r}_{23}$ can be partially compensated by the angular parts of the remaining coordinates.

This set of basis states are convenient for handling the FS blocking on the trial wave functions in the **k**-space representation. In the **k**-space, the Suris tetron basis states read

$|n_1, n_2, n_3, m\rangle =$

$$\sum_{\mathbf{k}_1,\mathbf{k}_2,\mathbf{q}} \varphi_{n_1,m}(\mathbf{k}_1)\varphi_{n_2,-m}(\mathbf{k}_2)\,\varphi_{n_3,0}(\mathbf{q}) a^{\dagger}_{\mathbf{k}_1;\sigma} a^{\dagger}_{\mathbf{k}_2;-\sigma} a_{\mathbf{q},-\sigma} b^{\dagger}_{\mathbf{Q}-\mathbf{k}_1-\mathbf{k}_2+\mathbf{q};j} |FS\rangle, \qquad (8)$$

with the constraint $m_1 + m_2 + m_3 = 0$. The total angular momentum is zero. Here $|FS\rangle$ denotes the Fermi sea, which includes both the K and K' valleys; σ $(-\sigma)$ is the spin of an electron at the K (K') point of the conduction band; $a^{\dagger}_{\mathbf{k};\pm\sigma}(a_{\mathbf{k},\pm\sigma})$ creates (annihilates) an electron with wave vector **k** and spin $\pm\sigma$; $b^{\dagger}_{\mathbf{k};j}$ creates a valence hole with wave vector **k** and spin j. The electron-hole generation needs to obey the selection rules of the optical transition between the valence and conduction bands. $\varphi_{n_1,m_1}(\mathbf{k}_1)$ $[\varphi_{n_2,m_2}(\mathbf{k}_2)]$ is the Fourier transform of $f_{n_1,m_1}(\mathbf{r}_{1h})$ $[f_{n_2,m_2}(\mathbf{r}_{2h})]$ in Supplementary Eq. (6), but truncated for $k_1 < k_F$ ($k_2 < k_F$) due to the FS blocking. We have

$$\varphi_{nm}(\mathbf{k}) = 2\pi W_n^{|m|}(k)\frac{1}{\sqrt{A}}e^{im\theta_k}\ , \qquad (9)$$

where $W_n^{|m|}(k) = S_n^{|m|}\frac{1}{k^{|m|}}\left(-\frac{\partial}{\partial\alpha_n}\right)^2\left[\frac{(f(\alpha_n)-\alpha_n)^{|m|}}{f(\alpha_n)}\right]$ for $|m| > 0$ and $W_n^{|m|}(k) = S_n^0\alpha_n/f^3(\alpha_n)$ for $m = 0$ with $f(\alpha) = \sqrt{\alpha^2 + k^2}$. $S_n^{|m|}$ is a normalization constant. $\varphi_{n_3,m_3}(\mathbf{k})$ is chosen to be a series of sinusoidal functions for k in the interval (0, k_F) multiplied by the angular function $e^{im\varphi_3}$ as given in Ref. 13.

In the calculation, the angular quantum number is restricted to $m_1, m_2 = 0, \pm1, \pm2$. The number of radial functions with different exponents (α_n) is chosen to be 10, which makes the size of the Hamiltonian matrix for the trion part to be 500. To describe the FS hole, we use ten sinusoidal basis functions $\varphi_{n_3,m_3}(\mathbf{k})$ (n_3 = 1, … 10) for each m_3 with $m_3 = 0, \pm1$. Although this set of basis functions are not complete, they are flexible enough to describe the low-lying states of the four-particle system and capture the main physics of the absorption and emission spectra. We have tested the performance of this set of basis on an ideal 2D system, and the calculated trion ground-state energy agrees well with the previous variational calculation[18] within 0.01 excitonic Rydberg for the electron-to-hole mass ratio from 0 to 1.

The Hamiltonian matrix elements within the set of basis functions can be evaluated numerically with good accuracy and efficiency via the same method described in Ref. 13. Due to the free-carrier screening in the doped sample, the interaction potential is modified to be:[19]

$$V_{sc}(q) = V_0(q)/\varepsilon(q) = \frac{e^2}{2\kappa\varepsilon_0[q(1+q\rho_0)+s(1-\sqrt{1-(2k_F/q)^2})/a_B]}. \qquad (10)$$

Here $\varepsilon(q) = 1 + sV_0(q)\frac{2\varepsilon_0 m_c}{\hbar^2}(1-\sqrt{1-(2k_F/q)^2}$ denotes a dielectric function due to free-carrier screening; s is the spin-valley degeneracy of monolayer $MoSe_2$; $V_0(q)$ is the statically screened Coulomb potential for charge-neutral monolayer $MoSe_2$; m_c is the effective carrier mass ($m_c = m_e$, m_h for electron and hole, respectively).

For monolayer $MoSe_2$ encapsulated by BN, the free carriers can only effectively screen the electric field within the $MoSe_2$ plane. Since a large portion of the electric field goes out of the $MoSe_2$ plane for the carrier-carrier Coulomb interaction, the free-carrier screening should be reduced, especially for exciton states with a large radius. To include such a reduction of free-carrier screening, we replace the dielectric function $\varepsilon(q)$ with

an effective dielectric function $\varepsilon_{eff}(q)$:

$$\varepsilon_{eff}(q) = (1-f) + f\varepsilon(q) = 1 + fsV_0(q)\frac{2\varepsilon_0 m_c}{\hbar^2}\left(1-\sqrt{1-(2k_F/q)^2}\right). \quad (11)$$

Here f denotes the fraction that the electric field lines for the carrier-carrier Coulomb interaction are within the $MoSe_2$ layer. In our model, f is a fitting parameter to match our data. The screened model potential therefore becomes $V_{sc}(q) = V_0(q)/\varepsilon_{eff}(q)$.

The electron-hole exchange interaction is represented by the $D(0)\delta(\mathbf{r})$ term given in Supplementary Eq. (3) (it is present only for the interaction between the $+\sigma$-spin electron and hole in the K valley). For the second carrier from the K' valley, which appears in the trion or exciton polaron, there is no electron-hole exchange interaction. This favors the binding of the trion or exciton polaron in monolayer $MoSe_2$. Without including this effect, the trion binding is ~16 meV, while including this effect, the binding energy increases to ~22 meV, which agrees much better with the experimental results.

7.3. Absorption spectra of exciton polarons

In our tetron model, the exciton polaron state (associated with 1s or 2s exciton) with a center-of-mass (CM) wave vector $\mathbf{Q}$ is represented by a linear combination of the bare exciton and Suris tetron in the following form:

$$\sum_{\mathbf{k}_1} C_{\mathbf{k}_1;\mathbf{Q}}\, a^{\dagger}_{\mathbf{k}_1;\sigma} b^{\dagger}_{\mathbf{Q}-\mathbf{k}_1;-\sigma}|FS\rangle + \sum_{\mathbf{k}_1,\mathbf{k}_2,\mathbf{q}} D_{\mathbf{k}_1,\mathbf{k}_2,\mathbf{q};\mathbf{Q}}\, a^{\dagger}_{\mathbf{k}_1;\sigma} b^{\dagger}_{\mathbf{Q}-\mathbf{k}_1-\mathbf{k}_2+\mathbf{q};-\sigma} a^{\dagger}_{\mathbf{k}_2;-\sigma} a_{\mathbf{q},-\sigma}|FS\rangle. \quad (12)$$

In the above expression, we only consider the spin-singlet configuration of the two electrons and neglect the spin-triplet configuration, because the exciton polaron becomes unbound for the triplet configuration. The inclusion of the triplet configuration has a very small effect on the exciton polaron states of interest here.

We need to calculate the two sets of coefficients $C_{\mathbf{k}_1;\mathbf{Q}}$ and $D_{\mathbf{k}_1,\mathbf{k}_2,\mathbf{q};\mathbf{Q}}$ in the exciton polaron state. Although they may be obtained by a brute-force method, it will be computationally intensive. Instead of doing a full brute-force calculation, here we adopt the Rayleigh-Ritz variational method by using a large basis set made of trial basis functions as described in Supplementary Eqs. (7, 8). This allows us to find the expansion coefficients $C_{\mathbf{k}_1;\mathbf{Q}}$ and $D_{\mathbf{k}_1,\mathbf{k}_2,\mathbf{q};\mathbf{Q}}$ efficiently for the low-lying (*e.g.* the lowest few hundreds) eigenstates of the four-particle system within a finite set of basis functions (~15000). Once these low-lying eigenstates are found, we can calculate the absorption spectra according to:[20]

$$A(\omega) \propto \sum_i \left|\sum_{\mathbf{k}_1} C_{\mathbf{k}_1;\mathbf{0}} \langle \tilde{c}_{\uparrow,\mathbf{k}_1}|\hat{\mathbf{e}}\cdot\mathbf{P}|\, v^h_{\uparrow,\mathbf{k}_1}\rangle\right|^2 / [(\hbar\omega - E_g - E_i)^2 + \gamma^2]. \quad (13)$$

Here E_i is the i-th eigenvalues of the model Hamiltonian for the tetron; E_g is the bandgap of monolayer $MoSe_2$; γ is a broadening parameter; $\mathbf{P}$ is the momentum operator; $\hat{\mathbf{e}}$ denotes the polarization of the incident light. The calculated absorption spectra for the exciton polarons associated with the A_{1s} state under electron (hole) injection are shown in Supplementary Fig. 7a (Supplementary Fig. 8a) (we use a reduction factor $f = 0.05$ for the free-carrier screening). The calculated absorption spectra

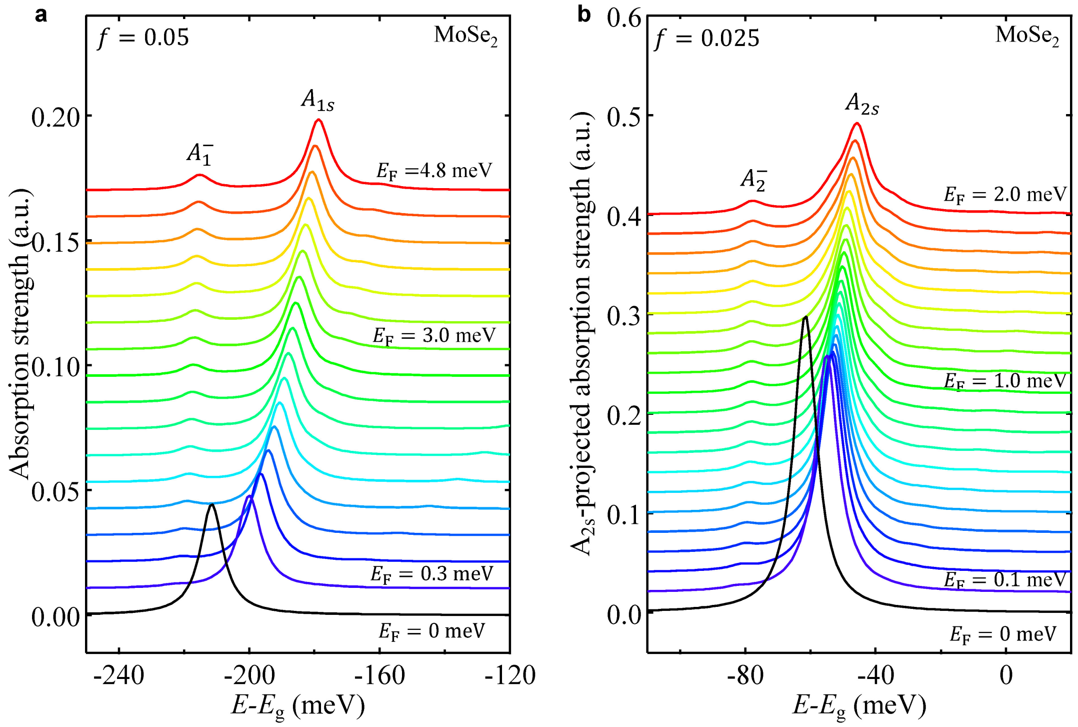

Supplementary Figure 7. Calculated absorption spectra of monolayer $MoSe_2$ at different Fermi levels on the electron side. a, Absorption spectra near A_{1s} calculated with f = 0.05. **b**, Absorption spectra near A_{2s} calculated with f = 0.025 after the projection onto the A_{2s} exciton orbital. The Fermi level (E_F) increases by 0.3 meV and 0.1 meV per spectrum in **a** and **b**, respectively. The x-axis ($E - E_g$) denotes the energy below the band gap (E_g).

for the exciton polarons associated with the A_{2s} state under electron (hole) injection are shown in Supplementary Fig. 7b (Supplementary Fig. 8b) (we use f = 0.025). A smaller f value is used for the 2s state because the exciton-polaron size is larger in the excited state than the ground state.

To make sure that the spectral features in the absorption spectra are associated with the A_{2s} state, we apply a projection operator on the calculated spectra to remove the background that is irrelevant to the A_{2s} state. The filtered spectra are plotted in Supplementary Fig. 7b. For the A_{1s}-related absorption, the results are qualitatively the same as those previously reported for 2D semiconductor quantum wells and 2D materials[13-17]. We note that our absorption calculation uses the long-range Coulomb interaction for both the electron-hole interaction within the exciton and the interaction between exciton and FS. Our model also considers the density-dependent carrier screening effect in the interaction potential and the FS blocking effect in the basis functions. This is more realistic than most prior theoretical works on exciton polarons[14-17], which use the contact potential to account for the interaction between exciton and FS, and do not consider the density dependence of carrier screening.

The calculated energy difference between the two lowest exciton-polaron peaks associated with the A_{1s} state as a function of gate voltage and Fermi level is shown as

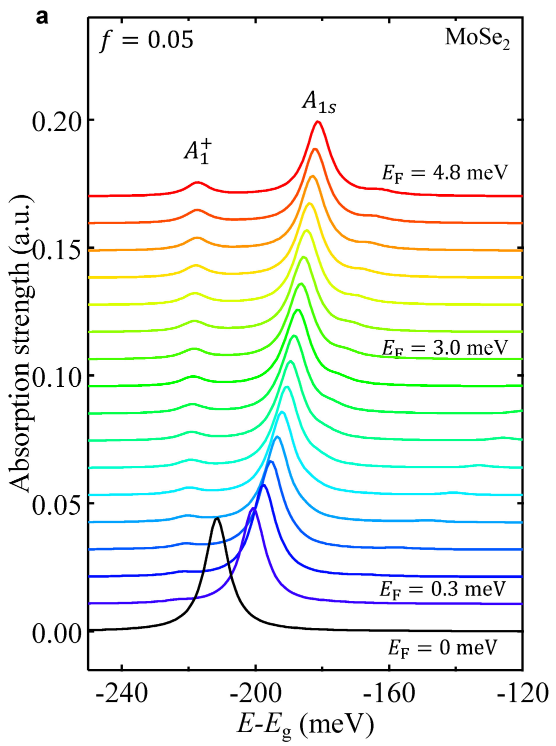

Supplementary Figure 8. Calculated absorption spectra of monolayer $MoSe_2$ at different Fermi levels on the hole side. a, **b**, Similar plots as in Supplementary Fig. 7 for hole injection. We use f = 0.05 and 0.025 in **a** and **b**, respectively.

the blue curve in Fig. 4d in the main paper. At low charge density, the lower peak is trion-like (with a loosely bound FS electron) and the higher peak is exciton-like (with a loosely bound electron and a FS hole). The dependence on the gate voltage agrees well with the experimental observation.

For the A_{2s}-related absorption spectra in Supplementary Fig. 7b, a resonance feature appears at ~19 meV below the A_{2s} peak and blueshifts gradually and almost linearly with increasing Fermi energy. The A_{2s} peak, suffering from the FS blocking and carrier screening effect, shows a sudden blueshift at low carrier concentration and then continues to blueshift gradually and almost linearly with increasing Fermi energy. The initial sudden blueshift is not observed in our experiment. It could be due to the lack of validity of the Thomas-Fermi screening theory at low carrier concentration, where the correlation effect is strong. The disagreement can also be attributed to the fact that the sample contains a certain amount of defects that can trap the carriers. At initial carrier injection, these localized traps get filled while no carrier screening and FS blocking are in effect. Only at a sufficiently high carrier density (when the traps are all filled), the carrier screening and blocking effects begin to set in. The evidence of these localized traps is also found in Ref. 21.

Thus, to compare the calculated energy difference between the resonance feature and the A_{2s} peak with the experimental data, we shall pay more attention to the behavior at higher carrier densities, where the carrier screening and FS blocking are in effect both

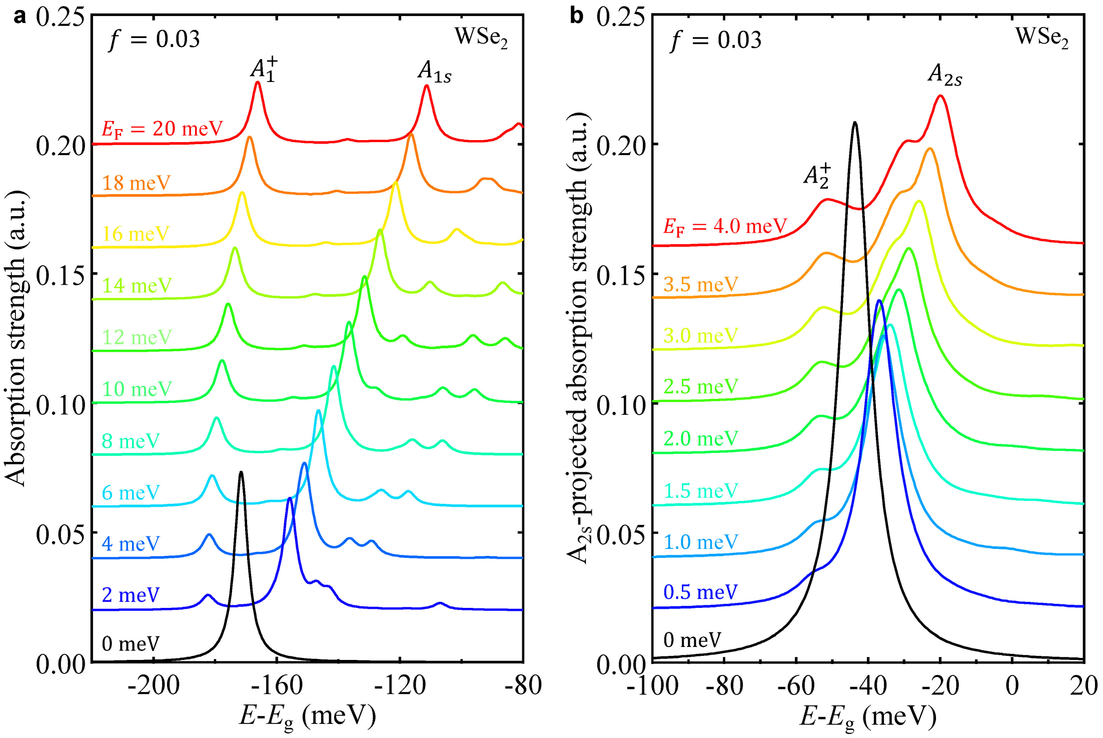

Supplementary Figure 9. Calculated absorption spectra of monolayer WSe$_2$ for different Fermi levels on the hole side. a, Absorption spectra near A_{1s} calculated with f = 0.03. **b**, Absorption spectra near A_{2s} calculated with f = 0.03 after the projection onto the A_{2s} exciton orbital. The Fermi level (E_F) increases by 2 meV and 0.5 meV per spectrum in **a** and **b**, respectively. The x-axis ($E - E_g$) is the energy below the band gap (E_g). Note that the double-bump feature for E_F beyond 3 meV for A_{2s} is caused by the finite number of Slater-type orbitals used to mimic the interaction of the A_{2s} exciton with the continuum.

theoretically and experimentally. We calculate the splitting energy between the two polaron peaks for E_F = 1.5 – 2 meV and use the linear interpolation to connect to the spliting energy at E_F = 0. The results are shown as the red lines in Figure 4d in the main paper. The agreement between theory and experiment is quite good. For low charge density, more thorough theoretical treatment that accounts for the influence of localized traps on the 2s-associted peaks is needed to fully explain the experimental observation.

We have also studied exciton polarons A_1^+ (A_2^+) associated with the A_{1s} (A_{2s}) excitons on positively charged monolayer WSe$_2$. Supplementary Figure 9 displays our calculated absorption spectra at different Fermi levels on the hole side. We note that the calculated splitting energy between the A_2^+ and A_{2s} resonance features agree well with our experimental data even without considering the effect of defects in the low charge density regime (E_F < 1.5 meV) as we have done for MoSe$_2$, presumably due to the better quality of WSe$_2$ crystals.

7.4. Summary of empirical parameters and experimental features explained

In our model, we have used the following empirical parameters:

(1) Exciton reduced effective mass (μ_X) and the characteristic length (ρ_0) in the Keldysh potential: We use $\mu_X = 0.4m_0(0.2m_0)$ and $\rho_0 = 1.07\text{nm}\,(1.17\text{nm})$ for monolayer $MoSe_2$ (WSe_2). The parameters are determined by fitting the measured energies and diamagnetic shifts of the exciton Rydberg states [1s-3s states for monolayer $MoSe_2$; 1s-4s states for monolayer WSe_2[8]].

(2) Electron and hole effective mass (m_e, m_h): We use $m_e = 0.88m_0\,(0.38m_0)$ and $m_h = 0.74m_0\,(0.42m_0)$ for monolayer $MoSe_2$ (WSe_2). These carrier masses are determined by the exciton reduced mass in (1) combined with the measured trion binding energy at $E_F = 0$ meV. The resultant carrier masses are consistent with the band structure calculated by the density function theory (DFT)[22] and Shubnikov–de Haas (SdH) measurements[20].

(3) *f* factors: We use $f = 0.05$ (0.03) for the 1s-associated exciton polaron and $f = 0.025$ (0.03) for the 2s-associated exciton polaron in monolayer $MoSe_2$ (WSe_2). For a monolayer semiconductor embedded in BN, a large fraction of the electric field between carriers goes out of the plane, leading to a reduction of in-plane free-carrier screening. The different f factors are used to account for the different screening reductions for the 1s and 2s states. The f factors are determined by fitting the E_F-dependent exciton-polaron binding energies in Figs. 4d and 5f in the main paper.

(4) Exciton polaron spectral width: For the absorption spectra, we use a constant broadening $\gamma = 4.2$ meV for both the 1s- and 2s-associated exciton polarons in monolayer $MoSe_2$ (Supplementary Figs. 7-8), and use $\gamma = 2.6$ meV (5.2 meV) for the 1s-associated (2s-associated) exciton polarons in monolayer WSe_2 (Supplementary Fig. 9).

By using the above empirical parameters, all of which are within reasonable physical range, our model can reproduce almost all of the essential experimental features for both the 1s- and 2s-associated exciton polarons in monolayer $MoSe_2$ and WSe_2, including:

1. The energy spacing of exciton Rydberg states
2. The diamagnetic shifts of the exciton Rydberg states
3. The E_F-dependent exciton-polaron binding energies and the energy spacing between the 1s- and 2s-associated exciton-polaron peaks.

Overall, the good agreement between theory and experiment strongly supports our conclusion of the exciton-polaron nature of the excitonic states in charged monolayer $MoSe_2$ and WSe_2.

Supplementary references

1. Hecht, E. Optics, Edn. 3rd Edition. (Addison-Wesley, New York; 1998).
2. Kuzmenko, A.B. Kramers–Kronig constrained variational analysis of optical spectra. *Rev. Sci. Instrum.* **76**, 083108 (2005).
3. Weber, J.W., Calado, V.E. & Sanden, M.C.M.v.d. Optical constants of graphene measured by spectroscopic ellipsometry. *Appl. Phys. Lett.* **97**, 091904 (2010).

4. Segura, A. et al. Natural optical anisotropy of h-BN: Highest giant birefringence in a bulk crystal through the mid-infrared to ultraviolet range. *Phys. Rev. Mater.* **2**, 024001 (2018).
5. Lee, S.-Y., Jeong, T.-Y., Jung, S. & Yee, K.-J. Refractive index dispersion of hexagonal boron nitride in the visible and near-infrared. *phys. stat. sol. (b)* **256**, 1800417 (2019).
6. Chen, S.-Y. et al. Luminescent emission of excited Rydberg excitons from monolayer WSe_2. *Nano Lett.* **19**, 2464-2471 (2019).
7. Goryca, M. et al. Revealing exciton masses and dielectric properties of monolayer semiconductors with high magnetic fields. *Nature Commun.* **10**, 4172 (2019).
8. Liu, E. et al. Magnetophotoluminescence of exciton Rydberg states in monolayer WSe_2. *Phys. Rev. B* **99**, 205420 (2019).
9. Molas, M.R. et al. Energy spectrum of two-dimensional excitons in a nonuniform dielectric medium. *Phys. Rev. Lett.* **123**, 136801 (2019).
10. Keldysh, L. Coulomb interaction in thin semiconductor and semimetal films. *JETP Lett.* **29**, 658 (1979).
11. Suris, R. A. Optical Properties of 2D Systems with Interacting Electrons SE-9. NATO Science Series Vol. 119, 111–124 (Springer, Netherlands, 2003).
12. Koudinov, A.V. et al. Suris tetrons: Possible spectroscopic evidence for four-particle optical excitations of a two-dimensional electron gas. *Phys. Rev. Lett.* **112**, 147402 (2014).
13. Chang, Y.-C., Shiau, S.-Y. & Combescot, M. Crossover from trion-hole complex to exciton-polaron in n-doped two-dimensional semiconductor quantum wells. *Phys. Rev. B* **98**, 235203 (2018).
14. Suris, R.A. et al. Excitons and trions modified by interaction with a two-dimensional electron gas. *phys. stat. sol. (b)* **227**, 343-352 (2001).
15. Efimkin, D.K. & MacDonald, A.H. Many-body theory of trion absorption features in two-dimensional semiconductors. *Phys. Rev. B* **95**, 035417 (2017).
16. Efimkin, D.K. & MacDonald, A.H. Exciton-polarons in doped semiconductors in a strong magnetic field. *Phys. Rev. B* **97**, 235432 (2018).
17. Sidler, M. et al. Fermi polaron-polaritons in charge-tunable atomically thin semiconductors. *Nature Phys.* **13**, 255 (2017).
18. Stébé, B. & Ainane, A. Ground state energy and optical absorption of excitonic trions in two dimensional semiconductors. *Superlattices and Microstructures* **5**, 545-548 (1989).
19. Stern, F. Polarizability of a two-dimensional electron gas. *Phys. Rev. Lett.* **18**, 546-548 (1967).
20. Larentis, S. et al. Large effective mass and interaction-enhanced Zeeman splitting of K-valley electrons in $MoSe_2$. *Phys. Rev. B* **97**, 201407(R) (2018).

21. Liu, E. et al. Landau-quantized excitonic absorption and luminescence in a monolayer valley semiconductor. *Phys. Rev. Lett.* **124**, 097401 (2020).
22. Jin, Z., Li, X., Mullen, J.T. & Kim, K.W. Intrinsic transport properties of electrons and holes in monolayer transition-metal dichalcogenides. *Phys. Rev. B* **90**, 045422 (2014).
